# Optimal Allocation of the Limited COVID-19 Vaccine Supply in South Korea

**DOI:** 10.3390/jcm10040591

**Published:** 2021-02-04

**Authors:** Eunha Shim

**Affiliations:** Department of Mathematics, Soongsil University, Seoul 06978, Korea; alicia@ssu.ac.kr; Tel.: +82-(2)-820-0416; Fax: +82-(2)-824-4383

**Keywords:** COVID-19, SARS-CoV-2, vaccine, Korea, vaccine allocation strategy, optimal strategy, mathematical model

## Abstract

Initial supply of the coronavirus disease (COVID-19) vaccine may be limited, necessitating its effective use. Herein, an age-structured model of COVID-19 spread in South Korea is parameterized to understand the epidemiological characteristics of COVID-19. The model determines optimal vaccine allocation for minimizing infections, deaths, and years of life lost while accounting for population factors, such as country-specific age distribution and contact structure, and various levels of vaccine efficacy. A transmission-blocking vaccine should be prioritized in adults aged 20–49 years and those older than 50 years to minimize the cumulative incidence and mortality, respectively. A strategy to minimize years of life lost involves the vaccination of adults aged 40–69 years, reflecting the relatively high case-fatality rates and years of life lost in this age group. An incidence-minimizing vaccination strategy is highly sensitive to vaccine efficacy, and vaccines with lower efficacy should be administered to teenagers and adults aged 50–59 years. Consideration of age-specific contact rates and vaccine efficacy is critical to optimize vaccine allocation. New recommendations for COVID-19 vaccines under consideration by the Korean Centers for Disease Control and Prevention are mainly based on a mortality-minimizing allocation strategy.

## 1. Introduction

The severe acute respiratory syndrome coronavirus 2 (SARS-CoV-2) has resulted in a public health and economic crisis, with more than 83 million confirmed infections and 1.8 million deaths globally due to the novel coronavirus disease (COVID-19) as of 5 January 2021 [1]. Various nonpharmaceutical interventions have been implemented, including the use of facemasks, handwashing, shelter-in-place orders, remote schooling, workplace closures, cancellation of mass gatherings, and travel restrictions, to reduce the mortality and morbidity burden of COVID-19. These nonpharmaceutical interventions are partially effective for reducing the disease burden, while more than 40 candidate vaccines are undergoing human trials and more than 150 vaccines are in the preclinical trial stage [2].

As of 5 January 2021, nine vaccines have been approved for public use by multiple countries: two messenger RNA (mRNA) vaccines (tozinameran from Pfizer–BioNTech and mRNA-1273 from Moderna), two viral vector vaccines (Gam-COVID-Vac from the Gamaleya Research Institute and AZD1222 from the University of Oxford and AstraZeneca), one peptide vaccine (EpiVacCorona from Federal Budgetary Research Institution State Research Center of Virology and Biotechnology), and four inactivated vaccines (BBIBP-CorV from Sinopharm, CoronaVac from Sinovac, Covaxin from Bharat Biotech and ICMR, Wuhan Institute of Biological Products, and an unnamed vaccine from the China National Pharmaceutical Group) [3]. Among these, two mRNA vaccine candidates developed by Pfizer–BioNTech and Moderna have recently shown approximately 95% efficacy in preventing laboratory-confirmed COVID-19 [4,5]. Based on a combined analysis, another vaccine candidate from AstraZeneca has shown approximately 70% efficacy in preventing symptomatic infection at or after 14 days following the second dose [6,7]. Specifically, the efficacy of the vaccine from AstraZeneca was 90.0% and 62.1% among those who received the lower-dose and full-dose vaccines, respectively, although the reasons for this difference are undetermined [6,7].

As of 8 January 2021, it was reported that 17.3 million doses of COVID-19 vaccines had been administered worldwide, including 5.9, 4.5, and 1.7 million doses in the US, China, and Israel, respectively [8]. The Korea Disease Control and Prevention Agency (KDCA) recently announced that they have secured enough COVID-19 vaccines to inoculate 56 million people, that is, more than enough to cover the country’s population of 52 million [9]. Specifically, South Korea has signed an agreement to purchase vaccines for 20 million people from Moderna, 10 million people from AstraZeneca, 6 million from Johnson & Johnson’s Janssen, and 10 million from Pfizer. In addition, the country has secured vaccines for 10 million people from the global vaccine supply platform, COVAX Facility [10].

Nevertheless, vaccine availability in the initial phases is likely to be limited. In South Korea, vaccine arrivals will begin in the first quarter of 2021, starting with the AstraZeneca vaccine, followed by Janssen and Moderna vaccines in the second quarter, and Pfizer vaccine in the third [11]. Furthermore, many factors related to potential vaccines are still unknown, including age-specific efficacy, duration of protection from disease, and effectiveness in subpopulations not evaluated in the clinical trials. Thus, it is imperative to develop optimal vaccination strategies to maximize the benefits of a vaccine for individuals and their communities under different supply and efficacy scenarios.

Past experience with delayed vaccine distribution in the mid-pandemic stage provides insights for the prioritization of COVID-19 vaccinations. During the novel A/H1N1 influenza pandemic of 2009 in the USA, several studies investigated optimal vaccination strategies for minimizing the disease burden [12,13,14,15,16]. These studies indicated that vaccinating school-aged children as well as their parents would be the optimal scenario when vaccine supply is limited in order to indirectly protect others in the community and, thus, reduce the number of severe health outcomes [12,13,15]. In the context of COVID-19 vaccination, Meehan et al. assumed two distinct modes of action for potential vaccines: those that protect against initial infection and those that reduce the severity of symptoms [17]. The study by Meehan et al. suggested that, in general, individuals aged 30–59 years should receive the highest priority for vaccination because of their high contact rates and high risk of infection [17]. However, Bubar et al. found that in the USA, a transmission-blocking vaccine should be prioritized in younger adults (age 20–49 years) to minimize the cumulative incidence and in people older than 60 years to minimize mortality [18]. Similarly, Matrajt et al. recommended vaccination of elderly people in the USA to minimize the number of deaths and hospitalization but recommended the vaccination of younger individuals (age 20–50 years) to minimize the cumulative number of infections, under the assumption that vaccine can reduce both the probability of acquiring infection and the probability of developing symptoms [19]. 

Our analysis examined the scenarios of the use a vaccine that confers partial protection to all the vaccinees, thereby reducing the probability of acquiring a SARS-CoV-2 infection. Specifically, we used updated information on vaccine availability and efficacy and examined the optimal vaccination strategies in South Korea, where COVID-19 control has been relatively successful. Compared to other countries where a less restrictive strategy was implemented, South Korea is one of the countries that implemented an early lockdown and used extensive testing and tracing [1]. Thus, South Korea has a relatively low incidence rate (123 per 100,000 population) compared to other countries (e.g., 6034 and 3982 per 100,000 population in the United States and France, respectively) [1]. Moreover, the cumulative deaths associated with COVID-19 per million population is relatively low in South Korea (estimated at 19) compared to the global average (~235), which is taken into consideration when we calculate country-specific optimal vaccination strategies for Korea [1]. 

Herein, we employ an age-structured dynamic model of COVID-19 transmission and vaccination to quantify the impact of COVID-19 vaccine prioritization strategies on health measures. Our model explicitly addresses variations in the supply of the COVID-19 vaccine and its protective efficacy. Furthermore, we consider country-specific characteristics in the population, such as age distribution, age-stratified contacts, and age-specific case-fatality rates. We identify and compare optimal vaccine allocation strategies in South Korea that minimize various healthcare measures, such as incidence, deaths, and years of life lost (YLL).

## 2. Experimental Section

### 2.1. Data

#### 2.1.1. Study Area and Population

We used nationwide data from Korea collected up to 26 November 2020, including information from all eight metropolitan/special cities and nine provinces. Age distribution and age-stratified contacts in South Korea were used to parameterize our mathematical model.

#### 2.1.2. COVID-19 Case Data

We obtained information on the cumulative number of confirmed cases and deaths up to 26 November 2020 from daily reports published by the Korea Centers for Disease Control and Prevention (KCDC) [9]. The data on confirmed cases and deaths associated with COVID-19 were stratified based on age (Table 1).

### 2.2. Mathematical Modeling

#### 2.2.1. Age-Structured Model of COVID-19 Transmission and Vaccination

**Figure 1:**
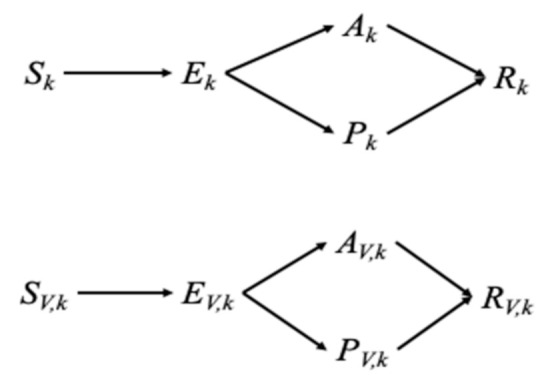
Movement of individuals between disease and vaccination status.

To model SARS-CoV-2 transmission and vaccination, we developed an age-structured model that incorporates five epidemiological compartments (i.e., susceptible, latent, asymptomatic and infectious, symptomatic and infectious, and recovered; Figure 1). Each epidemiological compartment is then subdivided into 16 age groups (0–4, 5–9, 10–14, …, 65–69, 70–74, and ≥75 years) and vaccination status. The dynamics of COVID-19 infection, illness, and infectiousness reflect our current understanding of the natural history of COVID-19. The unvaccinated individuals are divided into susceptible ($S_{k}$), exposed ($E_{k}$), asymptomatic ($A_{k}$), symptomatic ($P_{k}$), and recovered ($R_{k}$) categories. Similarly, vaccinated individuals are divided into susceptible ($S_{V,k}$), exposed ($E_{V,k}$), asymptomatic ($A_{V,k}$), symptomatic ($P_{V,k}$), and recovered ($R_{V,k}$) categories. Here, the subscript *k* refers to the age group of individuals (*k* = 1, 2, …, 16), while the subscript *V* indicates vaccinated groups. The full details of the mathematical model are provided in Appendix A.

#### 2.2.2. Force of Infection

The force of infection in age group *k*, $\lambda_{k}$, is expressed using a standard incidence formed in our model:

$$\lambda_{k}=\beta_{k}\sum_{j=1}^{16}c_{kj}\left(\frac{m(P_{j}+P_{V,j})+A_{j}+A_{V,j}}{N_{j}}\right),$$

where $\beta_{k}$ is the probability of a successful transmission among individuals in age group *k* after contact with an infectious individual, $c_{kj}$ is the number of age group-*j* individuals that an age group-*k* individual is in contact with per day, and *m* is the relative infectiousness among symptomatic individuals compared to that in asymptomatic individuals, estimated at 1.3 (see Table S1) [19,20]. Here, $N_{j}$ is the number of people in age group *j*, and thus $P_{j}/N_{j}$ and $A_{j}/N_{j}$ are the probabilities that a random age-*j* unvaccinated individual is infectious, with and without symptoms, respectively. We parameterized $c_{kj}$ using synthetic country-specific contact matrices that were constructed based on the POLYMOD study (Table S1) [20,21].

#### 2.2.3. Basic Reproductive Number

We further calculated the basic reproductive number (*R*_0_) using the next-generation operator method, where *R*_0_ is defined as the number of secondary cases caused by a single infective case in a completely susceptible population. Specifically, the expression of *R*_0_ can be derived as $R_{0}=\rho \left(FW^{-1}\right)$, which is the spectral radius of the next-generation matrix $FW^{-1}$ [15,22]. In our model, the next-generation matrix is determined by

$$FW^{-1}={\left(M_{kj}\right)}_{k,j=1,\ldots ,16}$$

where

$$\;M_{kj}=\beta_{k}c_{kj}\frac{S_{k}\left(0\right)}{N\left(0\right)}\left(\frac{a}{\gamma_{A}}+\frac{m\left(1-a\right)}{\gamma_{P}}\right)$$

Here, $\gamma_{A}$ and $\gamma_{P}$ are the infectious periods of asymptomatic and symptomatic individuals, respectively, with both estimated at 1/5 (day^−1^) [23,24]. We also define *a* as the proportion of infections that become asymptomatic, estimated at 0.35 [25,26]. We estimated the age-susceptibility $\beta_{k}$ to fit the relative incidence in age group *k* in South Korea and to obtain *R*_0_ = 2.6 (Table 1), consistent with recent estimates ranging from 2.1 to 3.1 [9,27,28].

#### 2.2.4. Vaccination and Initial Conditions

In this model, the vaccine is assumed to provide partial protection. Specifically, vaccinated individuals are assumed to become infected at a fraction $\left(1-\delta \right)$ of the rate at which unvaccinated susceptible individuals can become infected, where δ is the efficacy of the vaccine against infection. That is, we assume the use of a leaky vaccine, that is, a vaccine that confers partial protection to all the vaccinees, which can reduce the probability of acquiring the SARS-CoV-2 infection. Nevertheless, the impact of currently available COVID-19 vaccines on infection and, thus, transmission has yet to be further examined [29].

We assumed that unvaccinated people are initially susceptible and even vaccinated individuals might become infected at a reduced rate. In South Korea, the proportion of seropositive individuals is minimal at present (estimated at 0.07%) [30], and the rate of under-reporting is relatively low [31]. Specifically, the true number of cases exceeded the reported figure by a factor of 2.6, which is relatively low compared to those in other countries [31]. Therefore, we ignored the presence of seropositive individuals in the population when implementing vaccination strategies. Furthermore, we assumed that the antibodies are protective against reinfection.

#### 2.2.5. Calculation of Optimal Vaccination Strategies

We used a continuous-time age-structured ordinary differential equation (ODE) compartmental model. Across all scenarios considered, we simulated 1 year of dynamics and assumed that vaccine-induced immunity would last for the duration of the simulated time window. From the simulated results, we then derived the cumulative incidence and mortality, which were then used as outcomes when we compared the effects of vaccine prioritization strategies. We have determined the optimal age-specific distribution of a limited number of vaccine doses for three different health outcome measures and present our results in 10-year age categories. The first two health outcome measures were total infections averted and total deaths averted. We also used YLL, which weighs deaths by the expected remaining years of life for different ages. To calculate total YLL due to a death, we multiplied standard life expectancy (SLE) in age groups by the number of deaths (see the Supplementary Materials Figures S1 and S2 for details). As a baseline input parameter, we assumed that there would be a limited supply of vaccine doses, whereby only 50% of the Korean population could be vaccinated. To examine the sensitivity of our results to vaccine supplies, we changed the number of vaccine doses available and explored the scenarios where 10–90% of the Korean population could be vaccinated. We performed a sensitivity analysis with regard to vaccine efficacy and lower basic reproduction number, but the baseline vaccine efficacy against infection was taken as 70% unless stated otherwise [6,32]. Model equations were solved using the ODE45 solver and optimized using “fmincon” in Matlab version 9.3.0. (The MathWorks, Inc., Natick, MA, USA).

## 3. Results

In South Korea, the 20–29 years age group had the highest transmission incidence per capita, which indicates that younger adults are responsible for the majority of infection transmission (Table 1). However, the case–fatality rate is highest among the elderly (age ≥70 years; Table 1). These results highlight that the transmission dynamics of SARS-CoV-2 and optimal vaccine allocation must be explicitly considered in age groups.

**Figure 2:**
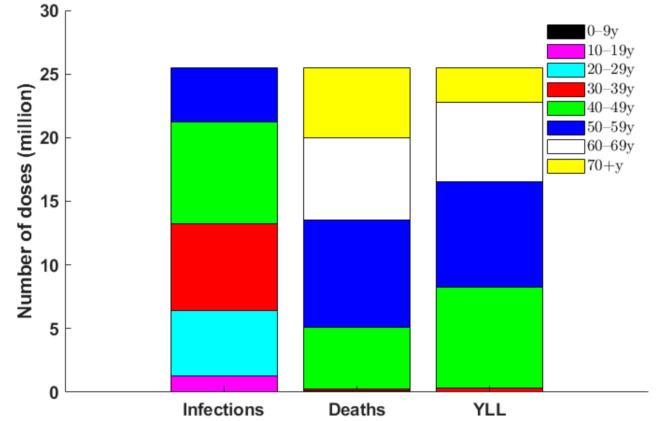
Number of vaccines optimally distributed to each age group from the 51 million available vaccine doses (i.e., doses to vaccinate 50% of the South Korean population) for each outcome measure.

**Figure 3:**
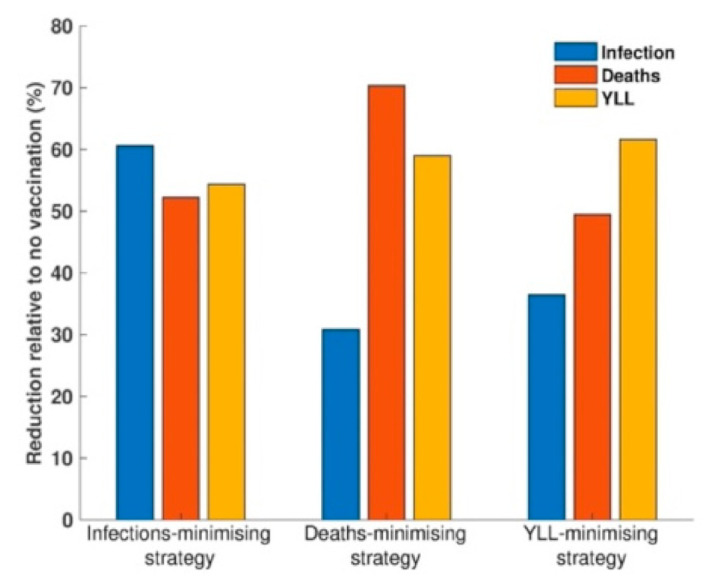
Comparison of outcome measures resulting from the allocation of 51 million vaccine doses for three different optimal vaccination strategies: to minimize the incidence, the number of deaths, and years of life lost (YLL).

**Figure 4:**
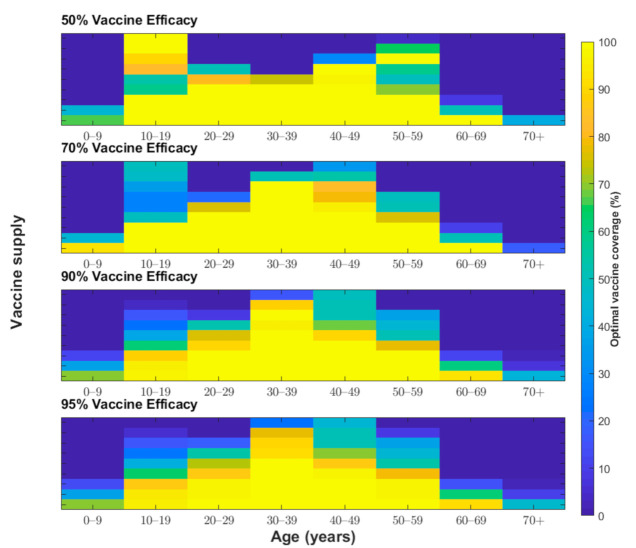
Proportion of individuals vaccinated from each age group (*x*-axis) to minimize the total number of infections under various vaccine coverage levels (*y*-axis) and vaccine efficacies (50%, 70%, 90%, and 95%).

**Figure 5:**
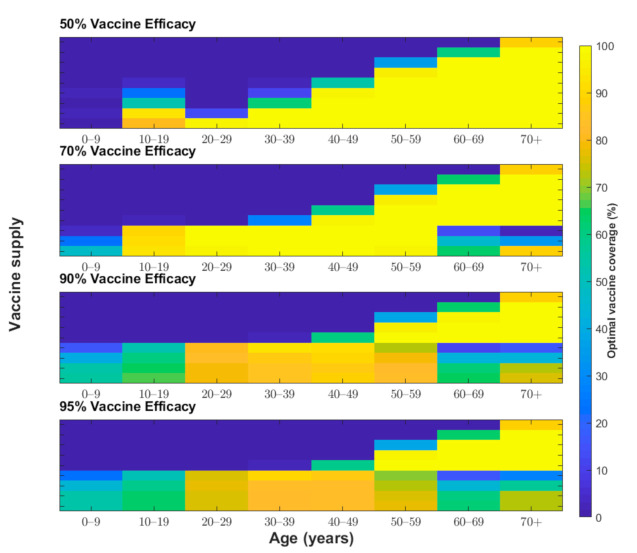
Proportion of individuals vaccinated from each age group (*x*-axis) to minimize the total number of deaths associated with COVID-19 under various vaccine coverage levels (*y*-axis) and vaccine efficacies (50%, 70%, 90%, and 95%).

**Figure 6:**
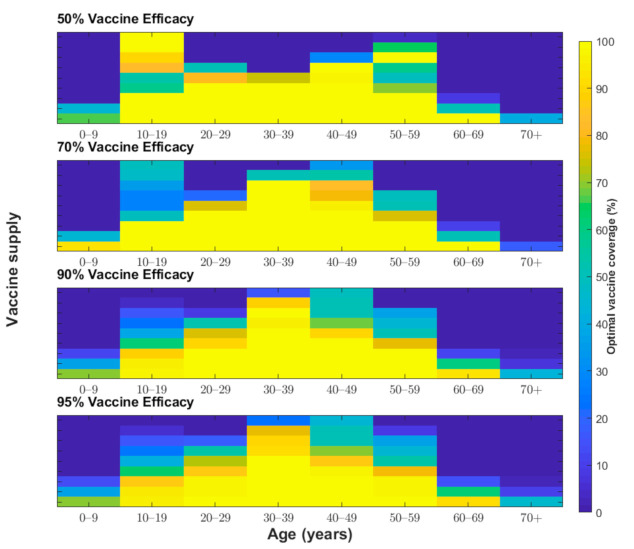
Proportion of individuals vaccinated from each age group (*x*-axis) to minimize the years of life lost under various vaccine coverage levels (*y*-axis) and vaccine efficacies (50%, 70%, 90%, and 95%).

We determined the optimal age-specific distribution of a range of available vaccine doses for three different outcome measures: incidence, deaths, and YLL. When 51 million vaccine doses are available to immunize 50% of the Korean population (with two doses per vaccine recipient) and vaccine efficacy is 70%, the incidence-minimizing strategy prioritized the vaccination of individuals aged 20–49 years to reduce the incidence by 61% (Figure 2, Figure 3 and Figure 4). However, to minimize mortality, the vaccines should be allocated to older adults aged 50 years and above, which would reduce total COVID-19 mortality by 70% across age groups (Figure 2, Figure 3 and Figure 5). The outcome measures of YLL are optimized by allocation to a slightly younger group—adults aged 40–69 years—because of the higher number of expected life-years remaining in this age group (Figure 2, Figure 3 and Figure 6).

We examined other scenarios with a lower number of vaccine doses than our baseline supply of 51 million. With less than 51 million vaccine doses and vaccine efficacy of 70%, the incidence-minimizing strategy switched to prioritizing those aged 10–19 years and 30–49 years due to active transmission between school-aged children and their parents. Moreover, the YLL-minimizing strategy narrowed its prioritization to adults aged 50–69 years due to a combination of the expected life years remaining and the greater infection severity in this age group. The mortality-minimizing strategy was optimized by vaccinating the elderly (age ≥70 years) and using the remaining doses for adults aged 60–69 years.

One of the most unclear characteristics of COVID-19 vaccines is their protective efficacy. The baseline scenario assumed a vaccine efficacy of 70%, which was chosen based on recent announcements by vaccine developers [6,32]. We considered two scenarios with higher vaccine efficacy (90% and 95%) and one more scenario with lower vaccine efficacy (50%) for our sensitivity analysis (Figure 4, Figure 5 and Figure 6). For a vaccine efficacy of 90% and 95%, the outcome measures of infections averted continued to be optimized by vaccine allocation to individuals aged 20–49 years, similar to that in the optimization based on a vaccine efficacy of 70% (Figure 4). Similarly, increased vaccine efficacy had a minimal effect on the mortality-minimizing strategy (Figure 5). However, using a higher vaccine efficacy resulted in the optimal vaccination strategy to minimize the YLL without a focus on elderly people; instead, the strategy included younger adults (age 30–59) due to better indirect protection (Figure 6).

The protective efficacy of COVID-19 in practice might be lower than that measured in clinical trial settings, and, thus, it is useful to be able to predict what might be the optimal strategy if vaccines have a reduced efficacy [33]. For the incidence-minimizing strategy, reducing vaccine efficacy against infection elevated the prioritization of vaccinating teenagers when the vaccine supply was increasingly limited. Moreover, assuming a lower vaccine efficacy resulted in the YLL-minimizing strategy switching from vaccinating individuals aged 30–59 years to vaccinating those in the ≥50-year age group. When the vaccine supply is very limited and the efficacy is low, individuals in the age group of 10–19 years and 50–59 years should be vaccinated in the optimal allocation strategy to minimize YLL. However, there was a minimal effect of reduced vaccine efficacy on optimal vaccine allocation to minimize deaths.

When the total supply is sufficiently high (i.e., ≥80%), incidence-minimizing strategies lead to a broader vaccination strategy for those aged 10–69 years; a small fraction of vaccines are prioritized for those aged 0–9 years. Generally, for mortality-minimizing and YLL-minimizing strategies, more vaccine doses were allocated to younger individuals with higher vaccine supplies, which relies on the indirect protection of vaccination. At a baseline vaccine efficacy of 70%, when the vaccine supply is abundant, the optimal allocation for all three strategies is mainly to vaccinate individuals aged 10–69 years; however, the mortality-minimizing strategy prioritized the vaccination of the oldest age group (≥70 years) over those aged 60–69 years.

**Figure 7:**
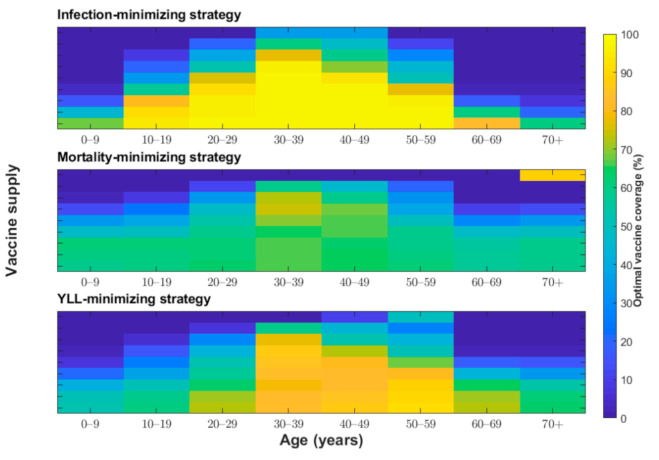
Impact of lower basic reproduction number on the proportion of individuals vaccinated from each age group (*x*-axis) to minimize three healthcare measures (i.e., total number of infections, total number of deaths, and the years of life lost) under various vaccine coverage levels (*y*-axis). Vaccine efficacy of 90% and a basic reproduction number (*R*_0_) of 1.5 are assumed.

We also analyzed the effects of a basic reproductive ratio of COVID-19 on the optimal control strategies of vaccination. Our results indicate that with a lower basic reproductive ratio (*R*_0_ = 1.5) and vaccine efficacy of 90%, the optimal vaccine allocation is generally focused on younger adults aged 30-49 years across all three aims (Figure 7). When the total supply is sufficiently high, all three optimal strategies lead to a broader vaccination strategy of those aged 10–69 years, with a small fraction of vaccines prioritized for those aged 0–9 and ≥70 years.

In order to examine more policy-relevant scenarios, we performed additional simulations under the assumption that none (0%) or complete (100%) vaccine coverage levels are allowed for each age group, with vaccine efficacy of 90% and two values of *R*_0_, i.e., *R*_0_ = 1.5 and *R*_0_ = 2.6 (Figures S1 and S2). Specifically, with a baseline basic reproduction number (*R*_0_ = 2.6) and 50% supply level, the incidence-minimizing strategy prioritized the vaccination of individuals aged 30–59 years, while the mortality-minimizing strategy and the YLL-minimizing strategy prioritized individuals aged 10–19 and ≥50 years, which indicates that the optimal strategy might vary depending on whether we allow partial vaccine coverage levels (Figure S1). On the other hand, with a lower basic reproduction number (*R*_0_ = 1.5) and 50% supply level, the incidence-minimizing strategy is to vaccinate individuals of ages 20–49, while the mortality-minimizing strategy and the YLL-minimizing strategies focuses on vaccinating individuals of age 30–59 years (Figure S2).

## 4. Discussion

Individuals at greatest risk of severe COVID-19 include the elderly and people with comorbidities. Vaccine allocation with limited supplies can be determined based on two rationales. First, vaccine distribution can be optimized to protect the population through direct protection, where high-risk groups, including elderly people, are prioritized to receive vaccines. Second, vaccines can be distributed to increase indirect protection, where individuals with high contact rates are vaccinated to reduce transmission. For instance, influenza vaccine campaigns initially relied on direct protection and, thus, targeted the elderly. However, influenza campaigns were modified to focus on the general population to enhance indirect protection rather than to protect the elderly, whose vaccine-induced immunity tends to be weaker than that of younger individuals [34].

This study demonstrates the use of a mathematical model of disease transmission and optimization process to evaluate vaccine prioritization strategies against COVID-19. Assuming 70% vaccine efficacy and a supply to immunize 50% of the population, we found that individuals in the 20–49 age group should be prioritized to minimize COVID-19 incidence. However, this recommendation is sensitive to vaccine efficacy because, with lower vaccine efficacy (30%), prioritization shifts toward teenagers and adults aged 50–59 years. COVID-19 vaccine candidates have recently shown at least 70% efficacy in clinical trials [6,32], and, thus, one can conclude that the vaccination target group for incidence-minimizing strategies would include individuals aged 20–49 years and then expand to neighboring age groups as more vaccine doses become available. In addition, it is known that the clinical characteristics and outcomes of COVID-19 patients are closely associated with age, with patients aged ≥60 years showing stronger clinical manifestations and greater severity compared with those aged <60 years, which necessitates the consideration of YLL associated with COVID-19 infection [35]. Optimal strategies to minimize YLL can exclusively target groups with high case-fatality rates (i.e., ≥50 years), thereby maximizing the direct benefit of vaccines when vaccine efficacy is relatively low (30%). However, with vaccines that have higher efficacy, prioritization shifts toward younger age groups: 40–69 years with 50–70% efficacy or 30–59 years with 90% efficacy. Thus, YLL-minimizing strategies are sensitive to vaccine efficacy and to the number of vaccine doses available.

In contrast, the mortality-minimizing strategies are relatively robust to variations in vaccine efficacy. Our modeling of mortality-minimizing strategies led to consistent recommendations to prioritize adults aged 50 years and older across efficacy values, in line with prior work [36]. This recommendation is robust because of the dramatic differences in case–fatality rates by age. Our model identified a few scenarios wherein prioritizing younger adults aged 20–59 years would provide greater mortality benefits than prioritizing older adults (age ≥50 years). These scenarios were restricted to the conditions of a large enough vaccine supply with high efficacy (≥70%). Thus, we conclude that with a limited supply of vaccine doses that is sufficient to immunize <50% of the population, the prioritization of vaccination in older adults is a robust strategy that is optimal to minimize mortality for all plausible vaccine efficacies; this is consistent with new recommendations that are under consideration by the Korean Centers for Disease Control and Prevention for COVID-19 vaccines.

Any age-based vaccination prioritization strategy, with various levels of vaccine supply and efficacy, can be evaluated using this model. Using our model, we have shown how prioritization can be optimized to meet the goals of minimizing health outcomes, such as incidence, YLL, and deaths, most efficiently. In summary, our approach shows that an incidence-minimizing strategy and a YLL-minimizing strategy prioritizes doses to individuals aged 20–49 and 40–69 years, respectively, whereas a mortality-minimizing strategy prioritizes direct vaccination of those older than 50 years. This finding is a consequence of the high case-fatality rates among the elderly and the ability of younger adults to induce indirect protection due to higher contact rates. However, our results on optimal strategies would be subject to the age-specific efficacy of vaccines, especially among elderly people who might show declining efficacy; this highlights the importance of age-stratified estimation of vaccine efficacy.

This study has several limitations. First, our study assumes that COVID-19 vaccines provide partial protection against acquiring infection. However, many factors related to these vaccines are currently unknown, including the impact of these COVID-19 vaccines on infection and, therefore, on transmission. The data from phase III trials suggest that all three vaccines, two mRNA vaccines (Pfizer-BioNTech and Moderna) and a DNA vaccine (Oxford-AstraZeneca), protect against symptomatic infection with SARS-CoV-2. However, sterilizing immunity in the upper airways was not proven, although challenge studies in vaccinated primates showed reductions in pathology, viral load, and symptoms in the lower respiratory tract [37,38]. Sterilizing immunity has been claimed for one protein-based vaccine manufactured by Novavax, although these data are yet to be published [29]. Furthermore, even if vaccines confer protection from disease, they might not similarly reduce transmission [29]. Second, our model assumes long-lasting vaccine-induced immunity, but it was suggested that vaccine-induced immunity might last only a few months [39]. If immunity is short-lived, then our results would be valid only for that time frame. Third, it is as yet unclear whether the first-generation of COVID-19 vaccines will be approved for individuals younger than 18 years [40]. This model assumes that individuals in all age groups would be eligible for vaccination; however, our approaches can be modified to evaluate the impact of vaccinating those within the age groups for which a vaccine is licensed. In addition, this study considers vaccine prioritization only by age, via age-structured contact matrices and age-specific susceptibility, although, for instance, a limited initial vaccine supply in the US and Canada is recommended to be allocated to healthcare or essential workers, long-term care facility residents, and adults aged 75 years or older [41,42]. Contact rates, as well as infection risk, vary not only by occupation and age but also by living arrangement and mobility, implicating the importance of considering spatial heterogeneity in infection potential. Last, YLL may be corrected for the comorbidities of the deceased, especially because COVID-19 frequently causes death in the elderly and those with underlying chronic conditions [43]. These limitations would be better addressed with further research on the risk factors of COVID-19 and population mobility during the pandemic.

Another factor that might be considered when determining vaccine prioritization would be the potential for vaccine hesitancy. Spatial heterogeneity may occur in vaccine hesitancy, which, when concentrated in a particular community or demographic group, would affect the vaccine acceptance rate, thereby hindering the administration of an optimal vaccination strategy. In the recent survey of 13,426 people in 19 countries, 71.5% of participants reported that they would take a COVID-19 vaccine if it were proven safe and effective, whereas 61.4% would accept their employer’s recommendation to do so [44]. Furthermore, if vaccine efficacy duration differs between age groups, then vaccine distribution strategies would need to be readjusted.

An age-structured mathematical model of COVID-19 transmission has facilitated the determination of the optimal allocation of vaccine doses for a variety of health measures. In our model, the policy objectives that were optimized were limited to a set of metrics of health outcomes, such as minimizing expected cases, YLL, and deaths. However, other social aspects, such as work productivity and returning to school, can be optimized. Moreover, it is unknown whether the vaccine candidates prevent the transmission of SARS-CoV-2 or mainly protect against illness. With the latter, it is unlikely that herd immunity would be achieved through immunization, necessitating changes in optimal vaccination strategies. A detailed evaluation of vaccine performance could help resolve these issues and improve model predictions and vaccine recommendations, thus providing a promising direction for future research.

## Figures and Tables

**Table 1:** Distribution of the cases by sex and age groups (as of 26 November 2020) [9].

		Confirmed Cases, *n* (%)	Deaths, *n* (%)	Fatality Rate (%)
Total		32,318 (100.00)	515 (100.00)	1.59
Age Group	≥80	1353 (4.19)	256 (49.71)	18.92
	70–79	2515 (7.78)	167 (32.43)	6.64
	60–69	4987 (15.43)	62 (12.04)	1.24
	50–59	5865 (18.15)	24 (4.66)	0.41
	40–49	4499 (13.92)	4 (0.78)	0.09
	30–39	4217 (12.77)	2 (0.39)	0.05
	20–29	6165 (19.08)	0 (0.00)	-
	10–19	1882 (5.82)	0 (0.00)	-
	0–9	925 (2.86)	0 (0.00)	-

## Data Availability

Publicly available datasets were analyzed in this study. This data can be found here: https://www.cdc.go.kr/board/board.es?mid=a30402000000&bid=0030.

## Supplementary Materials

The following are available online at https://www.mdpi.com/2077-0383/10/4/591/s1, Table S1: Contact matrix for South Korea; Table S2: Baseline parameter values and description; Table S3: Years of life lost in age groups due to death; Figure S1: The proportion of individuals vaccinated from each age group (*x*-axis) to minimize three healthcare measures (i.e., total number of infections, total number of deaths, and the years of life lost) under various vaccine coverage levels (*y*-axis) when only none (colored in blue) or complete (colored in yellow) vaccine coverage level is allowed for each age group, given a basic reproduction number (*R*_0_) of 2.6; Figure S2: The proportion of individuals vaccinated from each age group (*x*-axis) to minimize three healthcare measures (i.e., total number of infections, total number of deaths, and the years of life lost) under various vaccine coverage levels (*y*-axis) when only none (colored in blue) or complete (colored in yellow) vaccine coverage level is allowed for each age group, given a basic reproduction number (*R*_0_) of 1.5.

## Appendix A

To model the transmission of the severe acute respiratory syndrome coronavirus 2 (SARS-CoV-2) and the strategies for vaccination against the coronavirus disease (COVID-19), an age-structured model that incorporates five epidemiological compartments (i.e., susceptible, latent, asymptomatic and infectious, symptomatic and infectious, and recovered) was developed. Each epidemiological compartment was then subdivided into vaccination status and 16 age groups (0–4, 5–9, 10–14, …, 65–69, 70–74, and ≥75 years). The subscripts *U* and *V* represent the unvaccinated and vaccinated populations, respectively. In addition, $S_{U,k}\left(t\right)$, $E_{U,k}\left(t\right)$, $A_{U,k}\left(t\right)$, $P_{U,k}\left(t\right)$, and $R_{U,k}\left(t\right)$ represent the number of unvaccinated, susceptible, latent, asymptomatic and infectious, symptomatic and infectious, and recovered individuals, respectively, in age groups *k* at time *t* (*k* = 1, 2, …, 16). Vaccinated individuals are similarly divided into five categories ($S_{V,k}\left(t\right)$, $E_{V,k}\left(t\right)$, $A_{V,k}\left(t\right)$, $P_{V,k}\left(t\right)$, and $R_{V,k}\left(t\right)$), according to their epidemiological status. The dynamics of COVID-19 infection, illness, and infectiousness reflect the current understanding of the natural history of COVID-19 (Table S2). The proposed model for determining COVID-19 transmission and vaccination is described below.

$$\frac{dS_{k}}{dt}=-\lambda_{k}\left(t\right)S_{k}\left(t\right),$$

$$\frac{dE_{k}}{dt}=\lambda_{k}\left(t\right)S_{k}\left(t\right)-\sigma E_{k}\left(t\right),$$

$$\frac{dA_{k}}{dt}=a\sigma E_{k}\left(t\right)-\gamma_{A}A_{k}\left(t\right),$$

$$\frac{dP_{k}}{dt}=\left(1-a\right)\sigma E_{k}\left(t\right)-\;\gamma_{P}P_{k}\left(t\right),$$

$$\frac{dR_{k}}{dt}=\gamma_{A}A_{k}\left(t\right)+\;\gamma_{P}P_{k}\left(t\right),$$

$$\frac{dS_{V,k}}{dt}=-\lambda_{k}\left(t\right)\left(1-\delta \right)S_{V,k}\left(t\right),$$

$$\frac{dE_{V,k}}{dt}=\lambda_{k}\left(t\right)\left(1-\delta \right)S_{V,k}\left(t\right)-\sigma E_{V,k}\left(t\right),$$

$$\frac{dA_{V,k}}{dt}=a\sigma E_{V,k}\left(t\right)-\gamma_{A}A_{V,k}\left(t\right),$$

$$\frac{dP_{V,k}}{dt}=\left(1-a\right)\sigma E_{V,k}\left(t\right)-\;\gamma_{P}P_{V,k}\left(t\right),$$

$$\frac{dR_{V,k}}{dt}=\gamma_{A}A_{V,k}\left(t\right)+\;\gamma_{P}P_{V,k}\left(t\right),$$

where

$$\lambda_{k}=\beta_{k}\sum_{j=1}^{16}c_{kj}\left(\frac{m(P_{j}+P_{V,j})+A_{j}+A_{V,j}}{N_{j}}\right).$$

We assumed that people are initially susceptible unless they have been effectively vaccinated or have acquired natural immunity. We parameterized the initial conditions as follows:

$$S_{k}\left(0\right)=N_{k}\left(1-\varphi_{k}\right)-I_{k}\left(0\right),$$

$$S_{V,k}\left(0\right)=N_{k}\varphi_{k},$$

where for an age group *k*, $\varphi_{k}$ is the proportion of people of age group *k* vaccinated according to a given vaccination strategy. For optimization, we used three different objective functions: total symptomatic infections, total deaths, and total years of life lost (YLL). To calculate the YLL due to a death in age groups, we multiplied the standard life expectancy (SLE) in age groups by the number of deaths. Specifically, the country-specific SLE estimates from the World Health Organization Global Health Observatory was used in the calculations (Table S3) [45]. For the optimization process, we ran the deterministic model for 1 year and evaluated the optimal vaccine allocation for various combinations of vaccine efficacy and coverage levels (vaccine efficacy ranging from 30% to 90% in increments of 20%, and vaccination coverage ranging from 10% to 90% of the total population in increments of 10%).
