# Supplementary material for: Optimal Allocation of the Limited COVID-19 Vaccine Supply in South Korea
Source: J Clin Med. 2021 Feb 4;10(4):591. doi: 10.3390/jcm10040591 (PMC7914460; doi:10.3390/jcm10040591)
Supplement: Supplementary file 1 [file jcm-10-00591-s001.pdf]

Table S1. Contact matrix for South Korea [1,2].

1.323032	0.511391	0.259967	0.148988	0.207897	0.434599	0.722043	0.73318	0.453782	0.223322	0.250269	0.289737	0.224733	0.160106	0.086703	0.118455
0.511391	4.543004	1.296112	0.291484	0.160489	0.306496	0.786627	0.946801	0.858648	0.461342	0.455997	0.430193	0.22795	0.218478	0.180238	0.148089
0.259967	1.296112	9.382169	2.154623	0.288266	0.222286	0.572666	0.848998	1.147851	0.69275	0.775687	0.42713	0.169306	0.188677	0.20818	0.224877
0.148988	0.291484	2.154623	12.46826	1.843834	0.725586	0.535118	0.718495	1.186661	1.463987	1.142457	0.595319	0.271671	0.116446	0.190762	0.178754
0.207897	0.160489	0.288266	1.843834	3.908624	1.756962	1.057056	0.854554	0.904312	1.043929	1.017573	0.605064	0.276449	0.140007	0.110589	0.088538
0.434599	0.306496	0.222286	0.725586	1.756962	3.324036	1.729974	1.321534	1.170599	0.977348	1.208761	0.931825	0.445202	0.203659	0.157439	0.093461
0.722043	0.786627	0.572666	0.535118	1.057056	1.729974	3.148143	1.850105	1.541383	1.151196	1.088541	1.010244	0.596358	0.358766	0.195683	0.168644
0.73318	0.946801	0.848998	0.718495	0.854554	1.321534	1.850105	3.190067	2.027397	1.363772	1.102881	0.796407	0.605342	0.383384	0.32024	0.190943
0.453782	0.858648	1.147851	1.186661	0.904312	1.170599	1.541383	2.027397	3.131828	1.641143	1.462379	0.849986	0.515796	0.34596	0.384166	0.222199
0.223322	0.461342	0.69275	1.463987	1.043929	0.977348	1.151196	1.363772	1.641143	2.274281	1.564004	0.794123	0.428194	0.212035	0.287178	0.278001
0.250269	0.455997	0.775687	1.142457	1.017573	1.208761	1.088541	1.102881	1.462379	1.564004	2.24648	1.146448	0.47515	0.253375	0.253019	0.282998
0.289737	0.430193	0.42713	0.595319	0.605064	0.931825	1.010244	0.796407	0.849986	0.794123	1.146448	1.643023	0.618193	0.324085	0.212747	0.177926
0.224733	0.22795	0.169306	0.271671	0.276449	0.445202	0.596358	0.605342	0.515796	0.428194	0.47515	0.618193	0.924387	0.363836	0.335684	0.125091
0.160106	0.218478	0.188677	0.116446	0.140007	0.203659	0.358766	0.383384	0.34596	0.212035	0.253375	0.324085	0.363836	0.812451	0.350153	0.166004
0.086703	0.180238	0.20818	0.190762	0.110589	0.157439	0.195683	0.32024	0.384166	0.287178	0.253019	0.212747	0.335684	0.350153	0.767796	0.226417
0.118455	0.148089	0.224877	0.178754	0.088538	0.093461	0.168644	0.190943	0.222199	0.278001	0.282998	0.177926	0.125091	0.166004	0.226417	0.369968

Each entry in the contact matrix (c_{kj}) corresponds to the number of individuals of age-group j that a person in age group i comes into contact with.

Table S2. Baseline parameter values and description.

Parameter	Description	Baseline values	Refs
β_k	Probability of a successful transmission after contact with an infectious individual in age group *k*	0.40 (*k* = 1 and 2); 0.38 (*k* = 3 and 4); 0.79 (*k* = 5 and 6); 0.86 (*k*=7 and 8); 0.80 (*k* = 9 and 10); 0.82 (*k* = 11 and 12); 0.88 (*k* = 13 and 14); 0.74 (*k* = 15 and 16).	Fitted
N_k	Number of people in age group *k*	$N_1 = 1.70 * 10^6, N_2 = 2.28 * 10^6$, $N_3 = 2.28 * 10^6, N_4 = 2.48 * 10^6$, $N_5 = 3.23 * 10^6, N_6 = 3.46 * 10^6$, $N_7 = 3.11 * 10^6, N_8 = 3.72 * 10^6$, $N_9 = 3.84 * 10^6, N_{10} = 4.35 * 10^6$, $N_{11} = 4.34 * 10^6, N_{12} = 4.18 * 10^6$, $N_{13} = 3.81 * 10^6, N_{14} = 2.67 * 10^6$, $N_{15} = 2.00 * 10^6, N_{16} = 3.55 * 10^6$.	[3]
m	Relative infectiousness among symptomatic individuals compared to asymptomatic individuals	1.3	[4]
$1/\sigma$	Mean duration of exposure to infectiousness	3 days	[5]
a	Proportion of infections that become asymptomatic	0.35	[6,7]
$1/\gamma_A$	Infectious period of asymptomatically infected individuals	5 days	[8,9]
$1/\gamma_P$	Infectious period of symptomatically infected individuals	5 days	[8,9]
δ	Vaccine efficacy	0.7	[10,11]

Table S3. Years of life lost in age groups due to death [12].

Age group (*k*)	Ages (years)	Expectation of life (years)
1	<4	82.7
2	5–9	78.6
3	10–14	73.6
4	15–19	68.7
5	20–24	63.7
6	25–29	58.8
7	30–34	54.0
8	35–39	49.1
9	40–44	44.3
10	45–49	39.5
11	50–54	34.8
12	55–59	30.2
13	60–64	25.8
14	65–69	21.4
15	70–74	17.2
16	75+	10.4

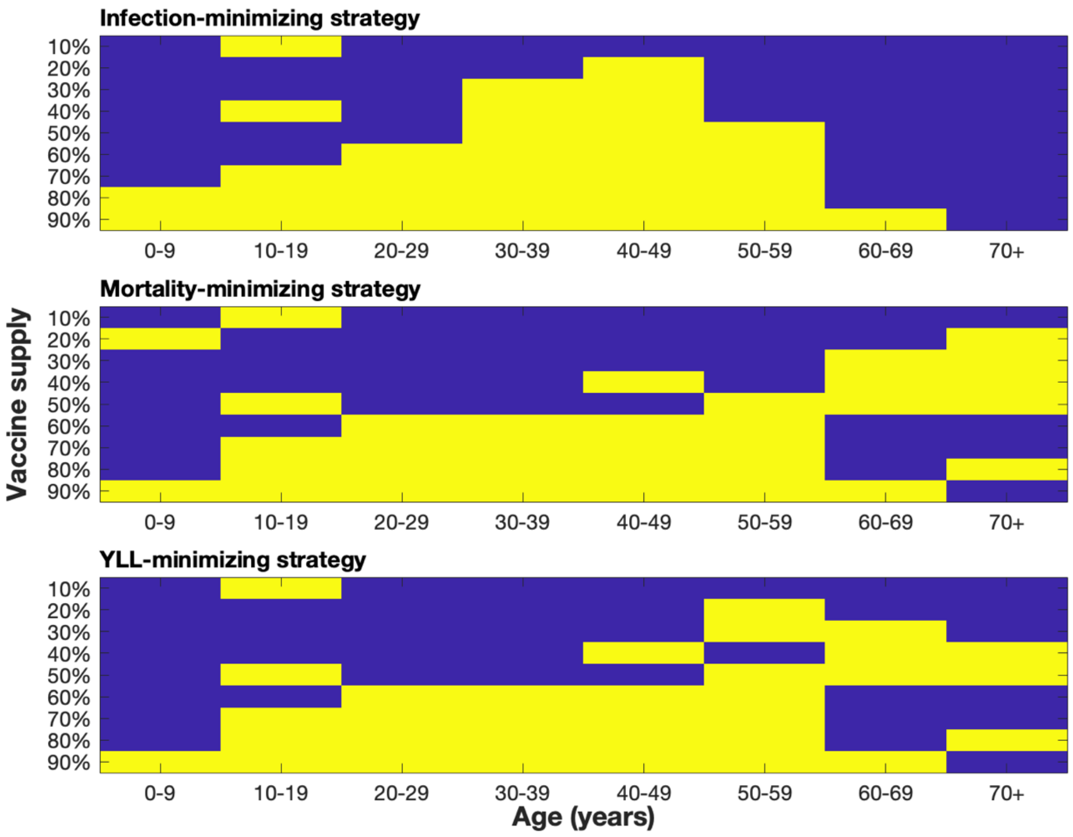

Figure S1. The proportion of individuals vaccinated from each age group (x-axis) to minimize three healthcare measures (i.e., total number of infections, total number of deaths, and the years of life lost) under various vaccine coverage levels (y-axis) when only none (colored in blue) or complete (colored in yellow) vaccine coverage level is allowed for each age group. Vaccine efficacy of 70% and a basic reproduction number (*R_0*) of 2.6 are assumed is assumed.

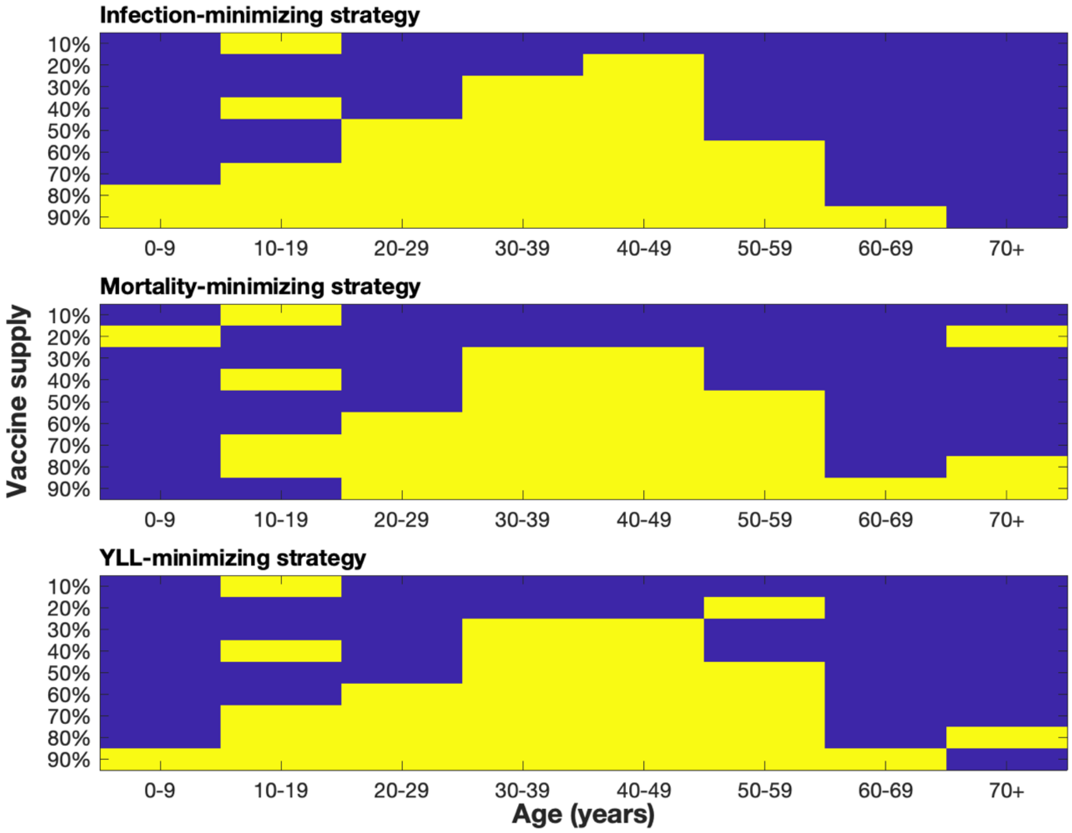

Figure S2. The proportion of individuals vaccinated from each age group (x-axis) to minimize three healthcare measures (i.e., total number of infections, total number of deaths, and the years of life lost) under various vaccine coverage levels (y-axis) when only none (colored in blue) or complete (colored in yellow) vaccine coverage level is allowed for each age group. Vaccine efficacy of 70% and a basic reproduction number (R_0) of 1.5 are assumed is assumed.
